# Anti-Hyperlipidemic Effects and Potential Mechanisms of Action of the Caffeoylquinic Acid-Rich *Pandanus tectorius* Fruit Extract in Hamsters Fed a High Fat-Diet

**DOI:** 10.1371/journal.pone.0061922

**Published:** 2013-04-16

**Authors:** Xiaopo Zhang, Chongming Wu, Haifeng Wu, Linghui Sheng, Yan Su, Xue Zhang, Hong Luan, Guibo Sun, Xiaobo Sun, Yu Tian, Yubin Ji, Peng Guo, Xudong Xu

**Affiliations:** 1 Pharmacology and Toxicology Research Center, Institute of Medicinal Plant Development, Chinese Academy of Medical Sciences, Peking Union Medical College, Beijing, China; 2 National Institute of Metrology, Beijing, China; 3 Research Centre on Life Sciences and Environment Sciences, Harbin University of Commerce, Harbin, China; National Research Council of Italy, Italy

## Abstract

Hyperlipidemia is considered to be one of the greatest risk factors contributing to the prevalence and severity of cardiovascular diseases. In this work, we investigated the anti-hyperlipidemic effect and potential mechanism of action of the *Pandanus tectorius* fruit extract in hamsters fed a high fat-diet (HFD). The *n*-butanol fraction of the *P. tectorius* fruit ethanol extract (PTF-b) was rich in caffeoylquinic acids (CQAs). Administration of PTF-b for 4 weeks effectively decreased retroperitoneal fat and the serum levels of total cholesterol (TC), triglycerides (TG) and low density lipoprotein–cholesterol (LDL-c) and hepatic TC and TG. The lipid signals (fatty acids, and cholesterol) in the liver as determined by nuclear magnetic resonance (NMR) were correspondingly reduced. Realtime quantitative PCR showed that the mRNA levels of PPARα and PPARα-regulated genes such as *ACO*, *CPT1*, *LPL* and *HSL* were largely enhanced by PTF-b. The transcription of *LDLR*, *CYP7A1*, and *PPARγ* was also upregulated. Treatment with PTF-b significantly stimulated the activation of AMP-activated protein kinase (AMPK) as well as the activity of serum and hepatic lipoprotein lipase (LPL). Together, these results suggest that administration of the PTF-b enriched in CQAs moderates hyperlipidemia and improves the liver lipid profile. These effects may be caused, at least in part, by increasing the expression of PPARα and its downstream genes and by upregulation of LPL and AMPK activities.

## Introduction

The incidence of hyperlipidemia, a disorder of lipid metabolism, is currently increasing at a dramatic rate throughout the world. The close relationship between hyperlipidemia and cardiovascular diseases (CVD) has been well documented [1], [2]. The lipoprotein of LDL-cholesterol (LDL-c) has been deemed as the primary risk factor of atherosclerosis (AS) and coronary heart disease (CHD) [3], [4], and the elevated circulating levels of free fatty acids (FFA) and triglycerides (TG) shows an important impact on the AS and CHD [5], [6]. Therefore, modulating the dysregulation of lipid metabolism and decreasing the elevated levels of serum TC, TG and LDL-c are considered to be quite beneficial for the treatment and prevention of CVD [7].

Peroxisome proliferator-activated receptor alpha (PPARα) plays a pivotal role in regulation of lipid metabolism [8], [9], [10]. Activation of PPARα by ligands upregulates the expression of genes involved in fatty acid transport and oxidation, such as acyl-CoA oxidase (ACO) [11], [12], carnitine palmitoyl transferase 1 (CPT1) [13], [14], [15], fatty acid transport protein (FATP) [16], [17], hormone-sensitive lipase (HSL) [18], [19], and lipoprotein lipase (LPL) [20], [21]. By altering transcription of these genes, activated PPARα leads to increased breakdown of triglycerides and fatty acids, increased cellular fatty acid uptake, and reduced triglyceride and fatty acid synthesis. Among the PPAR-regulated genes, LPL encodes the rate-limiting enzyme for the hydrolysis of the TG core of circulating TG-rich lipoproteins, such as those found in chylomicrons and very low-density lipoproteins (VLDL) [22], [23], playing an important role in TG metabolism. LPL not only removes TG from VLDL and decreases its size but also serves as a bridge between the cell surface and lipoproteins and promotes the uptake of LDL by cells. Therefore, LPL contributes to the clearance of both VLDL and LDL from the serum [24]. It has been reported that the hypotriglyceridemic action of PPARα agonist fenofibrate results at least in part from induction of the expression and activity of lipoprotein lipase (LPL) [25], [26].

AMP-activated protein kinase (AMPK) is another key regulator of lipid metabolism, imposing profound influence on lipid oxidation, synthesis, and storage [27], [28], [29]. AMPK activation turns on ATP-generating mechanisms such as lipid oxidation while switches off energy-consuming processes like TG and protein synthesis. The phosphorylation at threonine (Thr-172) on the alpha-subunit of AMPK has been deemed as an index of activation of this kinase which in turn promotes the phosphorylation and inhibition of acetyl-CoA carboxylase (ACC), a critical enzyme for controlling fatty acid biosynthesis and oxidation [30]. AMPK has now been proposed as a major therapeutic target for obesity and obesity-linked metabolic disorders such as hyperlipidemia [27].

Currently, a number of synthetic hypolipidemic drugs are available and are effective but the associated side effects such as diarrhea, nausea, myositis and abnormal liver function severely handicap their application. Some patients are resistant to or are intolerant of conventional pharmacotherapy. Therefore, alternative approaches are eagerly needed, and plant-based therapies attract much interest, as they are effective in reducing lipid levels and show minimal or no side effects. The Pandanaceae family comprises approximately 700 species, and many of these are important economic crops and are used as folk medicine for the treatment for leprosy, bronchitis, measles, dermatitis and diabetes [31], [32], [33]. In South China, the native people ingest the fruit of *Pandanus tectorius* as a folk medicine for the treatment of hyperlipidemia. However, to our knowledge, no scientific study of the anti-hyperlipidemic effect of the *Pandanus tectorius* fruit has been reported.

In the present study, the anti-hyperlipidemic activity of the *n*-butanol fraction from the ethanol extract of *Pandanus tectorius* fruit (PTF-b) was evaluated in hamsters fed a high-fat diet. An integrated methodology of pharmacological, molecular biological and analytical chemical approaches (ultra-performance liquid chromatography/quadrupole time-of-flight mass spectroscopy (UPLC/Q-TOF-MS) and nuclear magnetic resonance spectroscopy (NMR)-based metabolomics) was utilized to investigate the anti-hyperlipidemic activity and the potential mechanism of action of the PTF-b and to identify its active components.

## Results

### Characterization of the caffeoylquinic acids (CQAs) composition of the PTF-b using UPLC/Q-TOF-MS/MS

**Figure 1:**
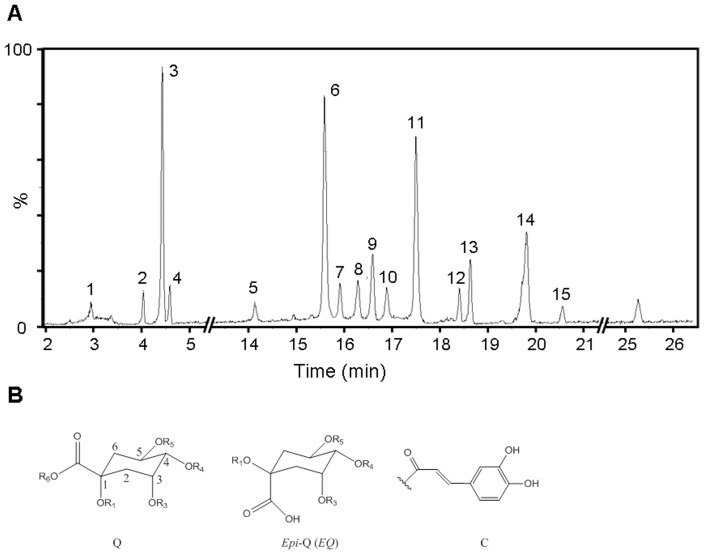
The primary caffeoylquinic acids present in the *P. tectorius* fruit extract PTF-b. (**A**) The total ion current profile corresponding to the UPLC-ESI-MS analysis of the PTF-b. (**B**) The skeleton of caffeoylquinic acid derivatives (IUPAC numbering). Peak numbers correspond to each of the caffeoylquinic acids (CQAs) identified in the PTF-b and are listed in Table S1.

UPLC/Q-TOF-MS/MS analysis revealed that the PTF-b was rich in caffeoylquinic acids (CQAs). Approximately twenty visible peaks could be determined from the total ion current profile of the PTF-b, of which fifteen were identified as caffeoylquinic acids. The compounds were characterized by their retention times and their UV and mass spectra and were identified by comparison with published data or commercial standards. A complete list with all the compounds identified in the PTF-b is shown in Table S1. The fifteen caffeoylquinic acids present in the PTF-b consisted of four caffeoylquinic acids (1-O-caffeoylquinic acid, 3-O-caffeoylquinic acid, 4-O-caffeoylquinic acid, and 5-O-caffeoylquinic acid), eight di-O-caffeoylquinic acids (1, 3-di-O-caffeoylquinic acid, 1, 4-di-O-caffeoylquinic acid, 1, 5-di-O-caffeoylquinic acid, 3, 4-di-O-caffeoylquinic acid, 3, 5-di-O-caffeoylquinic acid, 4, 5-di-O-caffeoylquinic acid, 1, 3-di-O-caffeoyl-*epi*-quinic acid, and 3, 5-di-O-caffeoyl-*epi*-quinic acid), two methyl esters of di-O-caffeoylquinic acids (methyl 1, 3-di-O-caffeoyl quinate and methyl 3, 4-di-O-caffeoyl quinate), and one tri-O-caffeoylquinic acid (3, 4, 5-tri-O-caffeoyl quinate). The 1, 4-di-O-caffeoylquinic acid, the 3, 4-di-O-caffeoylquinic acid, and the 3, 5-di-O-caffeoylquinic acid were designated as the major components of the PTF-b (Figure 1).

### The CQAs-rich PTF-b decreased the retroperitoneal fat accumulation and serum TC, TG and LDL-c levels in hamsters fed a high fat-diet

**Figure 2:**
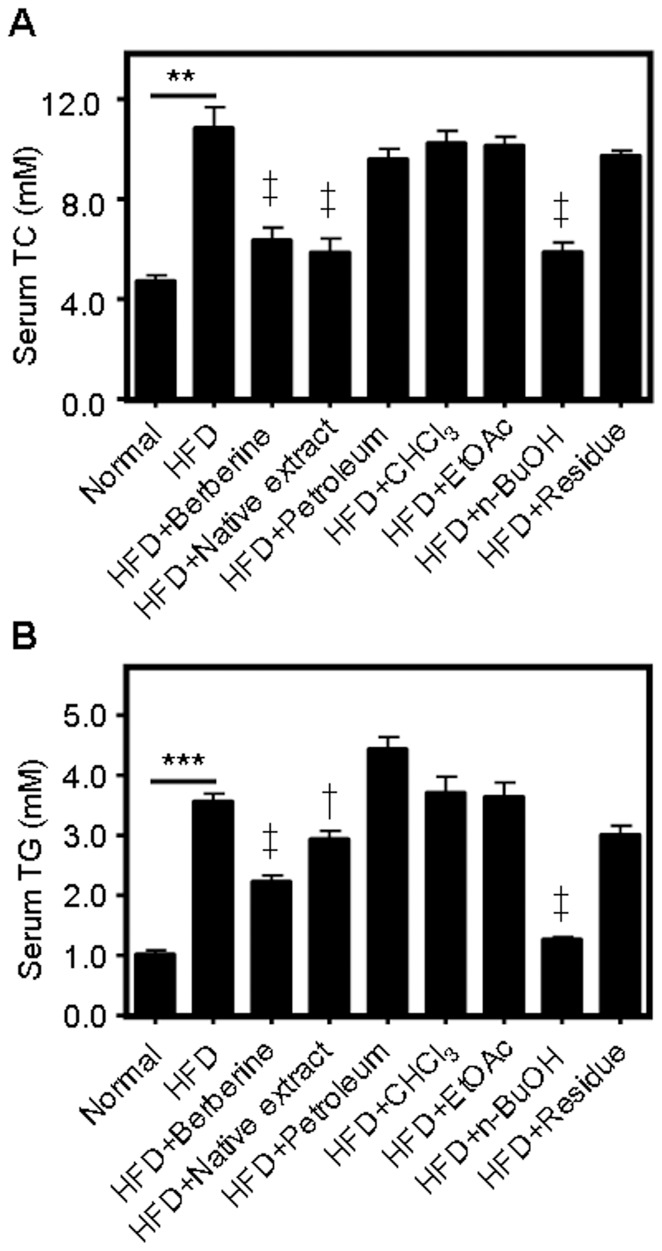
The effects of different fractions of *P. tectorius* fruit extract on the serum levels of total cholesterol (TC) (A) and triglycerides (TG) (B). The normal group was fed with a normal diet while the other groups were fed with a high fat-diet (HFD). The HFD+berberine and test groups (except the HFD+native extract group) were simultaneously supplemented with berberine (200 mg/kg) or individual fractions extracted from the native extract of *P. tectorius* fruit (100 mg/kg). The HFD+native extract group was treated with the 70% ethanol extract of *P. tectorius* fruit (500 mg/kg). **p<0.01, ***p<0.001 HFD group vs. normal group. ^†^ *p*<0.05, ^‡^ *p*<0.01 test group vs. HFD group.

Hamsters treated with a HFD for four weeks showed a significant increase in serum TC and TG (Figure 2). Supplementation with the 70% ethanol extract of *P. tectorius* fruit (native extract) largely reduced the blood levels of TC and TG with an efficacy comparable to berberine, a natural product widely reported to have anti-hyperlipidemic activities [34], [35], [36]. To identify the active fractions of *P. tectorius* fruit with blood lipid-lowering effects, the native extract was sequentially partitioned with petroleum ether, chloroform (CHCl3), ethyl acetate (EtOAc), and n-butanol (n-BuOH), and each fraction and the residue were further examined. As shown in Figure 2, the fractions of *P. tectorius* fruit extracted by petroleum ether, CHCl3, EtOAc and the remaining residue showed no effect on serum TC and TG, while the n-butanol fraction was quite effective in decreasing these blood lipids (Figure 2). This fraction was assigned as the PTF-b and investigated in detail for lipid modulatory activity.

**Figure 3:**
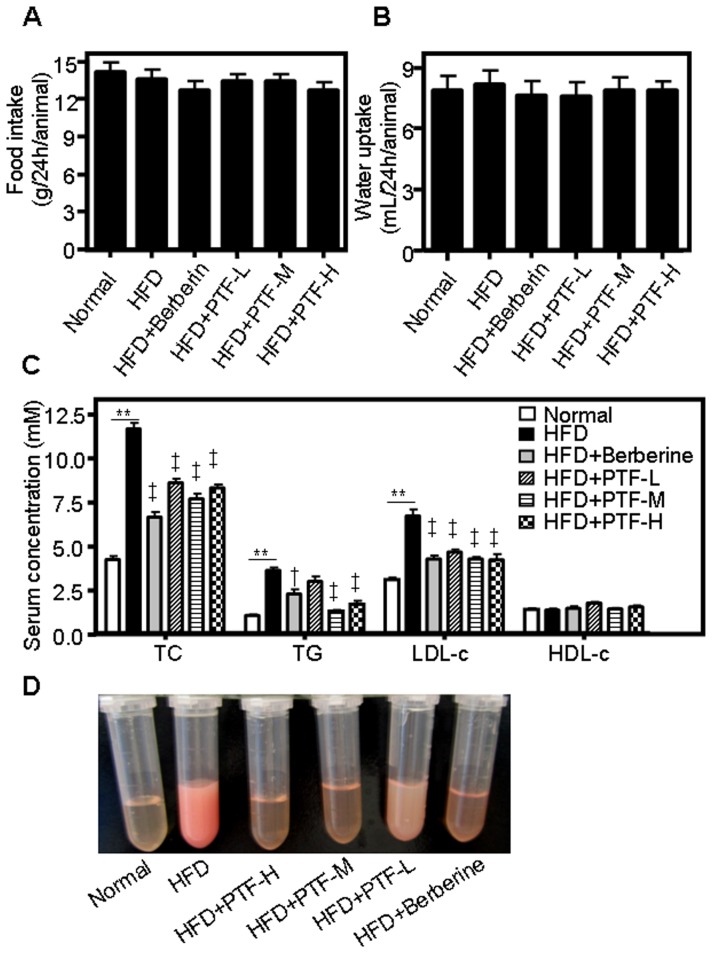
The CQA-rich PTF-b reduced serum total cholesterol (TC), triglycerides (TG) and low-density lipoprotein cholesterol (LDL-c) (C) without affecting food (A) and water (B) intake in hamsters. (D) Representative images of serum samples obtained from treated and untreated animals. The normal group was fed with a normal diet while the other groups were fed with a high fat-diet (HFD). The HFD+berberine and HFD+PTF-L, -M and -H groups were simultaneously supplemented with berberine (200 mg/kg) or the PTF-b at 50, 100 or 200 mg/kg, respectively. **p<0.01 HFD group vs. normal group. ^†^ *p*<0.05, ^‡^ *p*<0.01 test group vs. HFD group.

Feeding hamsters with a HFD for four weeks led to a significant gain of body weight and an increase in liver and retroperitoneal fat when compared with hamsters fed a normal diet (p<0.05, Table 1). Treatment with the PTF-b or berberine did not affect food or water consumption (Figure 3A and B) and showed no remarkable effect on body weight (Table 1). However, the weight of the retroperitoneal fat was significantly decreased in animals treated with berberine and the PTF-b. Correspondingly, the relative weight of the retroperitoneal fat compared with the whole body weight was significant lower in hamsters treated with 50 mg/kg of the PTF-b than in HFD control animals (0.0069±0.0005 vs. 0.0093±0.0017, Table 1).

**Table 1:** Effects of the PTF-b on the weight of body, liver and retroperitoneal fat in hamster (n = 10).

	Body weight (g)	Liver weight (g)	Retroperitoneal fat weight (g)	Liver/BW	Fat/BW
Normal	154.2±6.3	4.79±0.46	0.94±0.19	0.0310±0.0020	0.0061±0.0011
HFD	170.6±15.7*	8.14±1.29^**^	1.60±0.20*	0.0475±0.0032^**^	0.0093±0.0017*
HFD+Berberine	166.8±8.1	7.12±0.45	1.33±0.23^#^	0.0428±0.0029	0.0079±0.0012
HFD+PTF-b-L	169.0±11.5	7.57±0.84	1.17±0.13^##^	0.0448±0.0039	0.0069±0.0005^#^
HFD+PTF-b-M	162.0±5.2	7.06±0.47^#^	1.16±0.12^##^	0.0435±0.0022	0.0072±0.0009
HFD+PTF-b-H	172.5±15.6	7.69±0.98	1.32±0.28^#^	0.0445±0.0028	0.0076±0.0011

* *p*<0.05 HFD group Vs. Normal group, ^#^ *p*<0.05, ^##^ *p*<0.01 Test group Vs. HFD group.

As shown in Figure 3C, the serum levels of TC, TG and LDL-c in hamsters fed a HFD were increased more than two times over the normal control. Consistent with this, the color of the serum collected from HFD-treated hamsters appeared to be pale and turbid, while the serum from normal diet-treated animals was transparent and bright (Figure 3D). Supplementation with PTF-b significantly reduced the levels of serum TC, TG and LDL-c. At the optimal dose (100 mg/kg), PTF-b decreased the serum concentrations of TG by 64.51%, which was greater than the effect of berberine (200 mg/kg) (Figure 3C). At this dose, the inhibitory effect of PTF-b on LDL-c was similar with 200 mg/kg of berberine but the efficacy of PTF-b on TC was weaker than berberine. These data suggested that PTF-b was more potent in decreasing serum TG. The level of serum HDL-c was not affected by treatment with PTF-b or berberine.

### The CQAs-rich PTF-b inhibited lipid accumulation and decreased TC and TG levels in the liver

**Figure 4:**
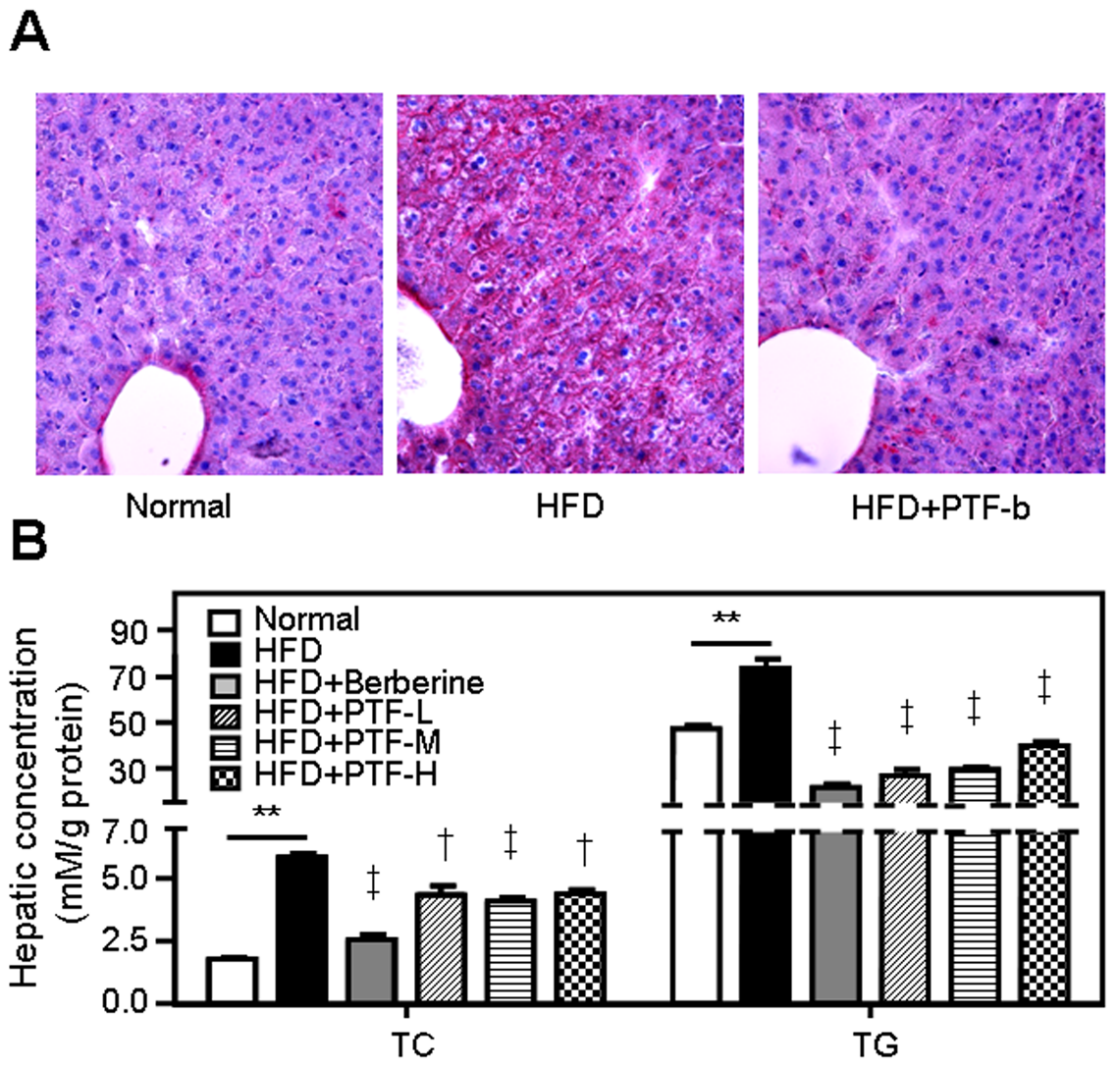
The CQA-rich PTF-b inhibited lipid accumulation and TC, TG levels in the liver. (**A**) The oil-red O staining pictures. (**B**) The hepatic TC and TG levels. The HFD+berberine and HFD+PTF-L, -M and -H groups were simultaneously supplemented with berberine (200 mg/kg) or the PTF-b at 50, 100 or 200 mg/kg, respectively. **p<0.01 HFD group vs. normal group. ^†^ *p*<0.05, ^‡^ *p*<0.01 test group vs. HFD group.

To examine whether supplementation with the PTF-b had beneficial effects on the levels of lipids in the liver, we first measured neutral lipid content in liver tissue using oil-red O staining. At the end of the experimental procedure, fat accumulation was markedly higher in HFD-fed hamsters than in normal diet-fed hamsters (Figure 4A). Following administration of the PTF-b (100 mg/kg), red staining representing neutral lipid accumulation was dramatically decreased. We further measured the levels of TG and total cholesterol in the liver and found that consumption of PTF-b markedly reduced the liver content of TC (25.88%, 30.14% and 24.91% by 50, 100 and 200 mg/kg PTF-b, respectively) and TG (63.72%, 60.01% and 46.11% by 50, 100 and 200 mg/kg PTF-b, respectively) (Figure 4B). At these doses, the efficacy of PTF-b in decreasing hepatic TC and TG was weaker than that of 200 mg/kg berberine especially in decreasing hepatic TC (Figure 4B).

### The CQAs-rich PTF-b increased LPL activity

**Figure 5:**
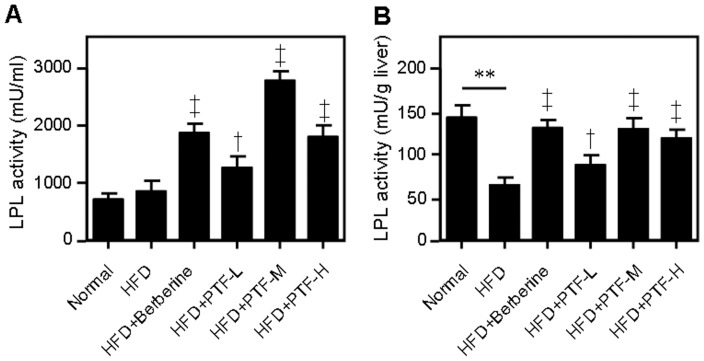
The CQA-rich PTF-b increased lipoprotein lipase (LPL) activity in serum (A) and liver (B). The normal group was fed with a normal diet while the other groups were fed with a high fat-diet (HFD). The HFD+berberine and HFD+PTF-L, -M and -H groups were simultaneously supplemented with berberine (200 mg/kg) or the PTF-b at 50, 100 or 200 mg/kg, respectively. **p<0.01 HFD group vs. normal group. ^†^ *p*<0.05, ^‡^ *p*<0.01 test group vs. HFD group.

Lipoprotein lipase (LPL) is the rate-limiting enzyme for the hydrolysis of triglyceride (TG)-rich lipoproteins. LPL activity has been demonstrated in a variety of tissues including lung, kidney, and liver [37], [38]. The dramatic decrease of serum and liver levels of TG by PTF-b promoted us to examine the effect of PTF-b on the activity of LPL. As shown in Figure 5, HFD did not change the serum activity of LPL but significantly decreased the LPL activity in liver. Administration of PTF-b largely increased the activity of LPL in serum and substantially reversed the decrease of hepatic LPL activity by HFD.

### The CQAs-rich PTF-b reversed some hyperlipidemia-related metabolites

**Figure 6:**
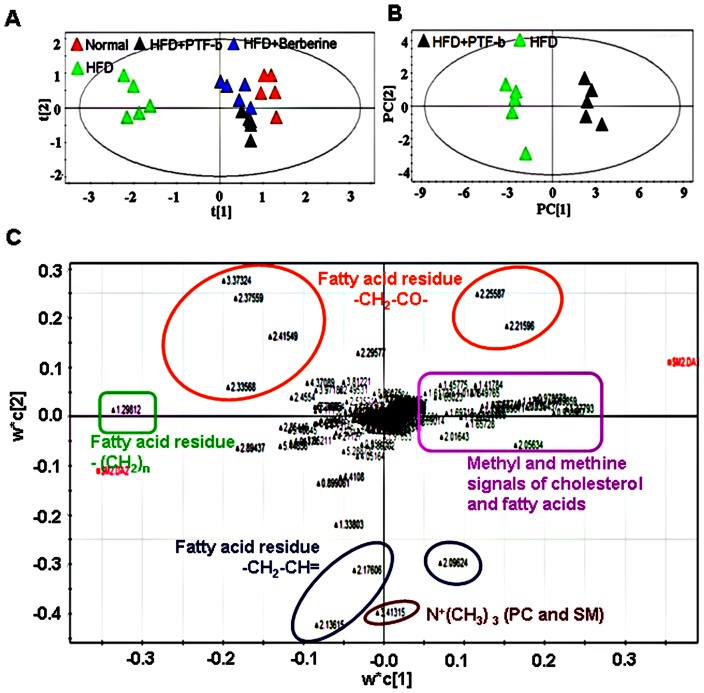
^1^H-NMR spectra of liver chloroform extract samples showed distinct separation between the HFD group and the HFD+PTF-b or HFD+berberine groups (A) Principal components analysis (PCA) of the ^1^H-NMR spectra of liver chloroform extract samples from normal and HFD-, HFD+berberine-, and HFD+PTF-b-treated hamsters. (B) Partial least squares-discriminant analysis (PLS-DA) score plots of the ^1^H-NMR spectra of liver chloroform extract samples showed obvious separation between HFD- and HFD+PTF-b-treated hamsters (R^2^X = 0.708, R^2^Y = 0.922, Q^2^ = 0.958). (C) PLS-DA loading plots of the ^1^H-NMR spectra of liver chloroform extract samples from HFD- and HFD+PTF-b-treated animals. Five types of lipids, *i.e*., fatty acid residues (-CH_2_-CH = , CH_2_-CH-, -CH_2_CH_2_CO-), methyl groups of cholesterol and fatty acids and N^+^(CH_3_)_3_ of PC and SM were significantly separated between the samples from the two groups.

NMR-based metabolomics has been shown to be an effective tool for investigating the pharmacological effects of drugs [39]. The liver tissues were subjected to NMR-based metabolomic analysis and the data were processed by principal components analysis (PCA) and partial least squares-discriminant analysis (PLS-DA) with the SIMCA-P software package. As shown in Figure 6A, the data points from the normal hamsters clustered to the right part of the plot whereas the data points from the HFD-fed animals localized to the far left part, with a distinct separation between the two groups. Supplementation with berberine or PTF-b caused the data to move towards the normal group, suggesting a remarkable normalization of lipid metabolism by these two reagents. The PLS-DA score plots (Figure 6B) also displayed an obvious separation between HFD and HFD+PTF-b groups with a high Q2 value of 0.958. The loading plots from the PLS-DA (Figure 6C) indicated that several NMR signals were significantly changed between the two groups. Analyzing these signals by visual comparison (Figure S1) and one-way ANOVA analysis (Figure S2) demonstrated that the metabolite profiles of the HFD+berberine and HFD+PTF-b groups contained lower levels of cholesterol, saturated and unsaturated fatty acids, and N+(CH3)3 (PC or SM) compared with that of the HFD group. These collective results demonstrate that the PTF-b effectively lowers the levels of cholesterol and fatty acids in the liver of hyperlipidemic hamsters, thus producing significant hypolipidemic effects.

### The PTF-b rich in CQAs upregulated the transcriptional expression of PPARα and activated AMPK in the liver

**Figure 7:**
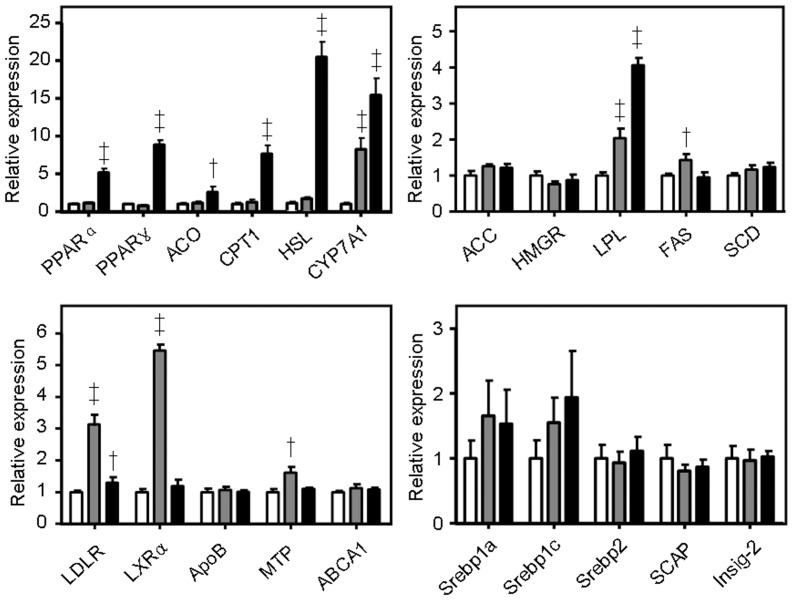
The CQA-rich PTF-b upregulated the transcriptional expression of LPL and PPAR-α and -γ in the liver. Real-time PCR was conducted with gene specific oligonucleotide primers as shown in Table S2. The amplification of β-actin served as the internal control. All groups were fed with a HFD. The HFD group (white) was treated with equal volume of distilled water, the HFD+berberine group (gray) was treated with 200 mg/kg of berberine and the HFD+PTF-b group (black) was treated with 100 mg/kg PTF-b. *p<0.05, **p<0.01 vs. HFD group.

To elucidate the potential mechanism of the anti-hyperlipidemic activity of the PTF-b, real-time quantitative PCR was first performed to determine the lipid metabolic genes whose expressions are regulated by the PTF-b. The hepatic mRNA levels of twenty-one lipid metabolism-related genes were quantified and compared between treated and untreated animals. As shown in Figure 7, supplementation with the PTF-b dramatically increased the mRNA levels of *PPARα* and *PPARα*-regulated genes such as *ACO, CPT1, HSL* and *LPL*, which indicated the upregulation of PPARα-related hypolipidemic pathway. The genes involved in LDL uptake (*LDLR*), cholesterol catabolism (*CYP7A1*), and insulin sensitivity (*PPARγ*) were also increased by PTF-b. The transcription of SREBP-1a and SREBP-1c was apparently up-regulated by PTF-b but the data were not significant due to the high variability of the analysis. The mRNA levels of SREBPs downstream genes such as *ACC, HMGR, FAS*, and *SCD*, and two SREBP-processing genes, SREBP cleavage-activating protein (*SCAP*) and insulin induced gene 2 (*Insig-2*), showed no significant change either after PTF-b treatment. These results indicated that PTF-b may decrease lipid accumulation mainly through upregulation of PPARα-related hypolipidemic pathway.

**Figure 8:**
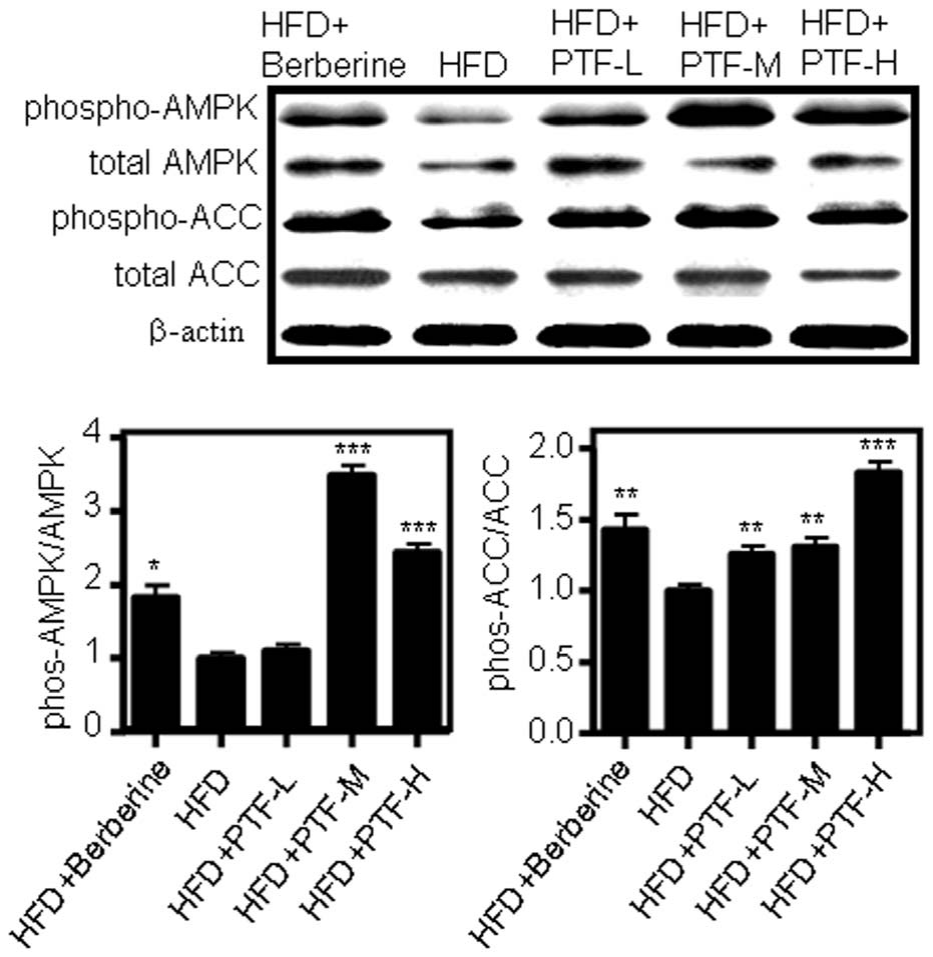
The CQA-rich PTF-b enhances AMP-activated protein kinase (AMPK) activation in the liver. Treatment with 100 mg/kg of the PTF-b for four weeks significantly increased the levels of phospho-AMPK and phospho-ACC in liver tissues. Protein levels were standardized against β-actin levels. The histograms in the lower panel represent the ratios of phospho-AMPK/total AMPK and phospho-ACC/total ACC, which represents the activation of AMPK. These values were obtained by quantification of the intensity of each band of the western blot. The normal group was fed with normal diet while the other groups were fed with a high fat-diet (HFD). The HFD+berberine group was treated with 200 mg/kg of berberine and the HFD+PTF-b-L, -M and -H groups were simultaneously supplemented with PTF-b 50, 100 or 200 mg/kg, respectively. *p<0.05, **p<0.01, ***p<0.001 vs. HFD group.

AMPK is a key regulator of lipid metabolism, playing a pivotal role in lipid oxidation, synthesis, and storage The hepatic protein levels of AMPK and its phosphorylated form (phospho-AMPK), which indicates the activation of AMPK, were evaluated by western blot. To confirm the activation of AMPK, the expression levels of ACC protein, a downstream target of AMPK, and its phosphorylated form (phospho-ACC) were simultaneously examined. As displayed in Figure 8, the PTF-b remarkably enhanced the levels of the activated form of AMPK (phospho-AMPK) and ACC (phosphor-ACC), which indicates the activation of AMPK by PTF-b.

### Caffeoylquinic acids (CQAs) may be the active components accounting for the anti-hyperlipidemic effect of the PTF-b

**Figure 9:**
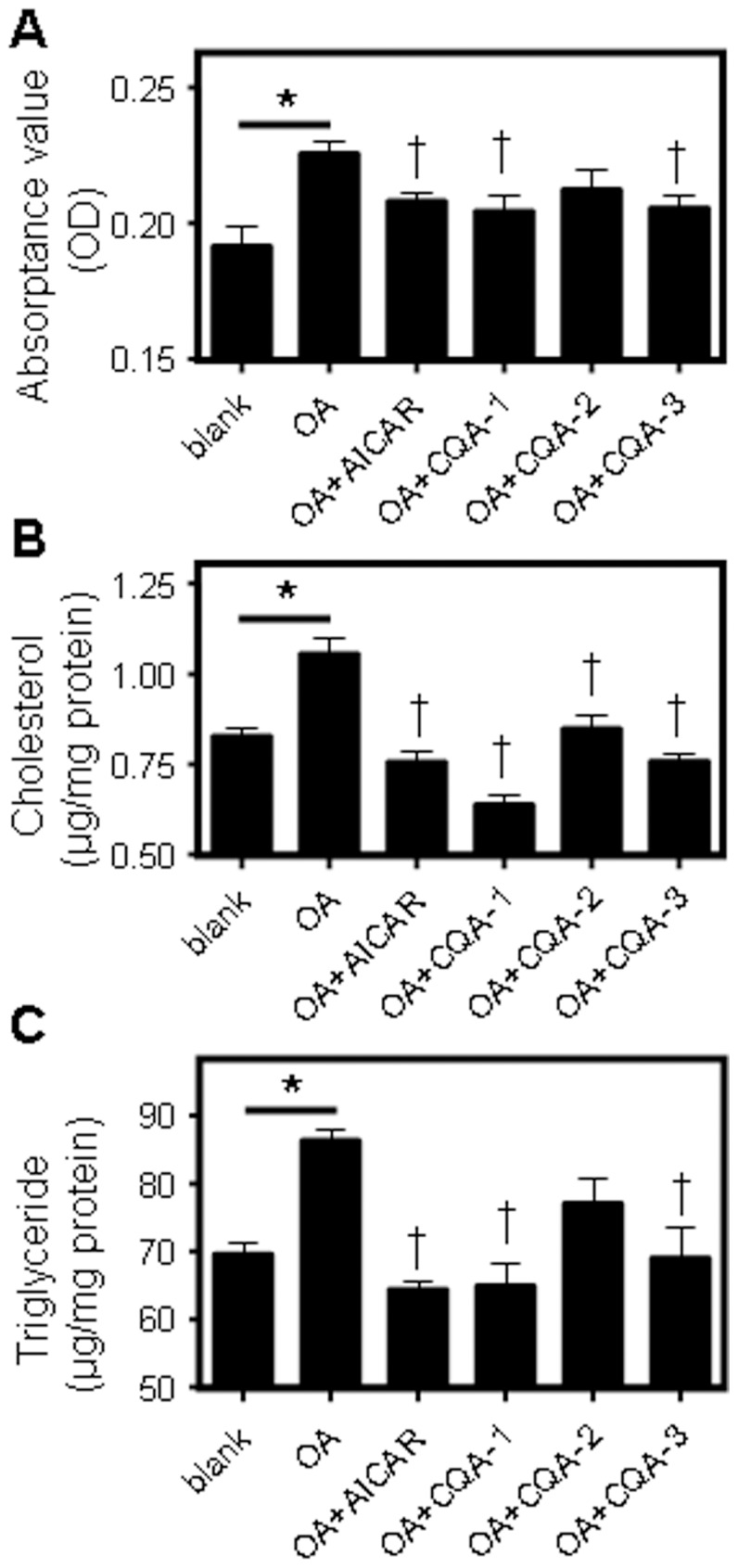
Three caffeoylquinic acids isolated from the PTF-b exhibited significant lipid-lowering effects in HepG2 cells. Cells were starved in 0.02% BSA/DMEM for 12 h and then incubated with 1 µM of the individual CQAs (3, 4-di-O-caffeoyl quinic acid (CQA-1), 3, 5-di-O-caffeoyl quinic acid (CQA-2) and 3, 4, 5-tri-O-caffeoyl quinic acid (CAQ-3)) or 1 mM AICAR in 0.02% BSA+100 µM oleic acid (OA)/DMEM or in 0.02% BSA+100 µM OA/DMEM alone for 6 h. Subsequently, the cells were subjected to oil-red O staining (**A**) or TC (**B**) and TG (**C**) determination. *p<0.05 OA group vs. normal group. ^†^ *p*<0.05 test group vs. OA group.

Phytochemical analysis showed that the PTF-b was very rich in caffeoylquinic acids (CQAs). To verify whether CQAs are the principal active components of the PTF-b accounting for the anti-hyperlipidemic activity, three CQAs which appear as large peaks in the total ion current profile (*i.e*., 3, 4-di-O-caffeoylquinic acid (peak 6, indicated as CQA-1), 3, 5-di-O-caffeoylquinic acid (peak 9, indicated as CQA-2) and 3, 4, 5-tri-O-caffeoylquinic acid (peak 14, indicated as CAQ-3)) were isolated from the PTF-b and tested for TC- and TG-lowering activities. As displayed in Figure 9, these three CQAs, particularly CQA-1 and CQA-3, significantly reduced the oil-red O staining (Figure 9A) and TC (Figure 9B) and TG (Figure 9C) accumulation in HepG2 cells, suggesting that CQAs play an important role in the anti-hyperlipidemic activity of the PTF-b.

## Discussion

In this study, we investigated for the first time the lipid regulatory activities of the ethanol extract of *P. tectorius* fruit as well as its n-butanol fraction (PTF-b) in hyperlipidemic hamsters induced by a high-fat diet (HFD). Through a combination of pharmacological, molecular and cell biological, and analytical chemical approaches, we demonstrated that the PTF-b possesses remarkable anti-hyperlipidemic activity which may work at least in part, by upregulating the expression of PPARα and its downstream genes and by promoting the activation of AMPK. The caffeoylquinic acids (CQAs) may be the active components accounting for the lipid modulatory effect of the PTF-b.

### PTF-b exhibits significant anti-hyperlipidemic effects in hamsters fed a HFD

We first assessed the anti-hyperlipidemic effects of the 70% ethanol extract of *P. tectorius* fruit and its *n*-butanol fraction (PTF-b) in HFD-fed hamsters, using the positive control berberine, which is a natural product well-documented to have the ability to reduce the blood and liver levels of TC and TG in various animal models [40], [41], [42]. Feeding with HFD for four weeks caused a large increase in serum and hepatic TC and TG in hamsters, manifesting in a severe hyperlipidemia. Treatment with berberine (200 mg/kg) substantially reduced the elevated levels of TC and TG in both the bloodstream and liver (Figure 2, 3C and 4B), which was in good accordance with previous reports [34], [43], [44], [45]. In animals fed a HFD, consumption of PTF-b (100 and 200 mg/kg) dramatically decreased the blood and hepatic levels of TC and TG (Figure 3C and 4B). This suggested that PTF-b is effective for the treatment of hyperlipidemia. In the dosage regimen we tested in this study, 50 mg/kg of PTF-b began to exhibit TC- and TG-lowering effects, and the optimal dose was determined to be 100 mg/kg.

The anti-hyperlipidemic activity of PTF-b was further confirmed by histochemical staining and NMR-based metabolomics. When sacrificed at the end of experiment, the bellies of the HFD-treated hamsters were filled with fat, and the livers appeared yellow (data not shown). Comparatively, the retroperitoneal fat accumulation was decreased and the liver appearance was significantly improved in the animals simultaneously supplemented with PTF-b. Treatment with 100 mg/kg of the PTF-b decreased retroperitoneal fat by 27.40% (Table 1) and markedly reduced the oil-red O staining of the liver, reversing it to a degree comparable to that of normal animals (Figure 4A). Analysis of the liver lipid profiles by NMR-based metabolomics also demonstrated that treatment with the PTF-b substantially reversed the disordered lipid metabolism of HFD-treated animals (Figure S1). The PTF-b showed a comparable lowering effect to berberine on liver unsaturated fatty acids (Figure S2A), cholesterol (Figure S2B), and PC and SM (Figure S2C), but was more effective in decreasing saturated fatty acids (Figure S2A). Therefore, the PTF-b is a promising agent for the treatment of hyperlipidemia.

### Caffeoylquinic acids (CQAs) may be the principle active components accounting for the anti-hyperlipidemic effect of the PTF-b

The total ion current profile of the PTF-b obtained by UPLC-Q-TOF/MS analysis showed that the PTF-b mainly consists of caffeoylquinic acids (CQAs) (Figure 1A), which account for 58% percent of the PTF-b by weight. It has been widely reported that the chlorogenic acid, one of the CQAs, from various plant species exhibit strong lipid-lowering effects in rodent models fed a HFD [46], [47], [48], [49], [50]. Administration of chlorogenic acid markedly decreased the concentration of TC, TG and free fatty acids (FFAs) in plasma and liver, which is most likely due to an increase in fatty acid utilization in the liver via the upregulation of PPARα mRNA [46], [48], [50]. The alteration of the activities of lipid metabolism enzymes such as hepatic lipase (HL), lipoprotein lipase (LPL) and 3-hydroxy 3-methylglutaryl coenzyme A (HMG-CoA) reductase (HMGR) are also involved in the anti-hyperlipidemic activities of chlorogenic acid [47], [48], [50], [51]. The beneficial effects of CQAs on hyperglycemia and hyperlipidemia have also been reported [52], [53]. Therefore, caffeoylquinic acids may be the active components of the PTF-b used for the treatment of hyperlipidemia.

To verify this hypothesis, we isolated three caffeoylquinic acids, namely 3, 4-di-O-caffeoylquinic acid (CQA-1), 3, 5-di-O-caffeoylquinic acid (CQA-2) and 3, 4, 5-tri-O-caffeoylquinic acid (CQA-3), from the PTF-b and tested their lipid-altering activities in HepG2 cells, using the AMPK activator 5-aminoimidazole-4-carboxamide 1-beta-ribofuranoside (AICAR) as a positive control [30]. Incubation with oleic acid (OA) dramatically increased fat deposition and cellular TC and TG content. Addition of the AMPK activator AICAR substantially inhibited the elevation of intracellular lipid concentration by OA (Figure 9), which was in good accordance with previous reports [30], [54]. After treatment with the three CQAs, particularly CQA-1 and CQA-3, neutral lipid accumulation in HepG2 cells, as determined by oil-red O staining, was significantly reduced (Figure 9A), and the intracellular content of TC and TG was correspondingly reduced (Figure 9B and 9C). These data demonstrated that the CQAs in the PTF-b exhibited a strong lipid-lowering activity in liver cells. Considering their potent lipid-modulating activity and their high concentration in the PTF-b, it is reasonable to conclude that caffeoylquinic acids (CQAs) may be the principle active component accounting for the anti-hyperlipidemic effect of the PTF-b.

### Upregulation of PPARα and AMPK may be involved in the anti-hyperlipidemic effect of PTF-b

The lipid metabolism in liver is regulated by a number of genes and proteins in which PPARα, AMPK, and SREBPs play central roles. PPARα increases the transcription of many genes that promote lipid hydrolysis and oxidation such as *LPL*, *HSL*, *ACO*, and *CPT1* [10], [18], [25]. AMPK phosphorylates and suppresses the activity of key proteins that promote lipogenesis such as SREBP1c [55], [56] and that inhibit lipid oxidation such as ACC [57], [58], [59]. On the contrary, SREBPs increase the synthesis of cholesterol, fatty acid, and triglyceride by enhancing the transcription of crucial genes involved in lipogenesis such as HMG CoA synthase (*HMGS*), HMG CoA reductase (*HMGR*), acetyl CoA carboxylase (*ACC*), fatty acid synthesis (*FAS*), and stearoyl CoA desaturase (*SCD*) [60]. It has been reported that the activities of SREBPs are regulated by *SCAP* and *Insig-2*, two regulators involved in SREBP processing and maturation [61], [62].

In this study, administration of PTF-b significantly enhanced the mRNA levels of PPARα and PPARα-regulated genes (*ACO*, *CPT1*, *HSL*) (Figure 7) and activated AMPK in a dose-dependent manner (Figure 8). The mRNA level and activity of LPL, the key enzyme in TG hydrolysis which is known to be upregulated by PPARα and AMPK [25], [26], were also increased (Figure 5 and 7). Treatment with PTF-b apparently up-regulated the transcription of SREBP-1a and SREBP-1c but the data were not significant due to the high variability of the analysis. The nuclear localization of SREBPs which indicates their activities was not determined in this study. Instead, we measured the mRNA levels of SREBPs-regulating (*SCAP* and *Insig-2*) and SREBPs-regulated genes (*ACC*, *HMGR*, *FAS* and *SCD*) and found that PTF-b showed no significant change on their expression (Figure 7). These results suggested that PTF-b may regulate lipid metabolism mainly through upregulation of PPARα and AMPK.

Although it has been reported that activation of PPARα led to increased expression of SREBPs and their downstream genes such as FAS, PTF-b significantly increased the expression of PPARα but it showed little influence on the expression of FAS. This may be due to the simultaneous stimulating effect of PTF-b on the activation of AMPK which is known to suppress the transcription of SREBPs. The balance between PTF-b induced PPARα and AMPK activation may result in the non-significant change in the expression of SREBPs and SREBPs-regulated genes in PTF-b treated animals. As PPARα, AMPK, and LPL play key roles in stimulating TG hydrolysis and fatty acid oxidation [59], [63], [64], the positive effects of PTF-b on PPARα, AMPK and LPL accounted, at least in part, for the TG-lowering role of PTF-b. AMPK activation also decreases cholesterol level in vivo [65]. PTF-b dramatically activated AMPK and increased the mRNA levels of LDLR and CYP7A1, two important genes in cholesterol uptake and catabolism [66], [67], which provided a potential mechanism for the cholesterol-lowering action of PTF-b.

Berberine was used as a positive control in this study which has been well-documented to exert hypolipidemic effects by upregulation of LDLR and activation of AMPK [34], [43], [44], [45]. As shown in Figure 7 and 8, berberine significantly stimulated LDLR expression and AMPK activation which were in accordance with previous reports. However, the gene expression profile in berberine-treated animals was not identical to that in PTF-b-treated ones. Although the regulatory effects of berberine on the expression of CYP7A1, LDLR, and LPL were similar with that of PTF-b, berberine showed little influence on the mRNA levels of PPARα and PPARα-regulated genes. These data indicated that the mechanism through which PTF-b and berberine decrease lipid accumulation was not identical though they both activate AMPK.

In summary, the combined approaches in present study proved to be effective not only in verifying the lipid-lowering effects of hypolipidemic herbal drugs used in the clinic or in folk medicine but also helpful in determining their potential mechanisms and their active components. Our findings suggest that the lipid-lowering effect of the PTF-b is due to the effect of CQAs in upregulating the transcriptional expression of PPARα and activation of AMPK in the liver. These promising findings provide incentive to find new alternative approaches for the treatment of hyperlipidemia. Therefore, further investigation of the interaction between compounds containing CQAs and their target effects are needed to generate a more complete understanding of the hypolipidemic effects of both the PTF-b and the natural products of the CQAs.

## Materials and Methods

### Ethics statement

All necessary permits were obtained for the described field studies. All animal experiments were approved by the Medical Ethics Committee of Peking Union Medical College and were in accordance with the National Institutes of Health regulations for the care and use of animals in research. All efforts were made to minimize suffering.

### Plant materials

The ripe fruits of *P. tectorius* were purchased from the public market in Hainan Province, P.R. China, in July 2011 and identified by Prof. Weiyong Lai at the School of Pharmaceutical Science, Hainan Medical University. A voucher specimen has been deposited there (NO. PT20110714). The PTF-b used in this study was prepared as described below. The dried fruits were exhaustively extracted with 70% ethanol and then filtered and concentrated under reduced pressure. The native extract was sequentially partitioned with petroleum ether, CHCl_3_, EtOAc and *n*-BuOH. The native extract, individual fractions and the residue were used for the evaluation of their anti-hyperlipidemic activities.

### Animals and experimental design

To evaluate the anti-hyperlipidemic activity of *P. tectorius* fruit and to determine the active fraction, animal experiments were performed. Seventy-two male Syrian golden hamsters (Vital River Laboratory Animal Technology Co., Ltd., Beijing, China) were kept in a humidity-controlled room on a 12-h light–dark cycle with food and water available *ad libitum* for one week. The animals were then divided randomly into nine groups with eight animals in each group. The normal group was fed with a normal diet while the other groups were fed with a high fat-diet (HFD). The normal rodent chow contained 12% fat, 62% carbohydrate, and 26% protein, with a total energy content of 12.6 kJ/g. The HFD was formulated to balance micronutrient content on a per calorie basis and contained 60% fat, 14% protein, and 26% carbohydrate, with total energy content of 21.0 kJ/g. The HFD contained much less choline bicitrate (0.6 g/kg) and DL-methionine (1.5 g/kg). Fatty acid composition of the fats (mainly from lard) in HFD was 36% saturated fatty acids, 45% monounsaturated fatty acids, and 19% polyunsaturated fatty acids (PUFA). The HFD+berberine and test groups (except the HFD+native extract group) were simultaneously supplemented with berberine (200 mg/kg) (Laboratoires Fournier S.A., Chenove, France) or individual fractions extracted from the native extract of *P. tectorius* fruit (100 mg/kg). The HFD+native extract group was treated with a 70% ethanol extract of *P. tectorius* fruit (500 mg/kg). At the end of the 4-week period, after the animals were fasted overnight, blood samples were collected for estimation of serum TC and TG (Jian Cheng Biotechnology Company, Nanjing, China).

In the study of the PTF-b, sixty male Syrian golden hamsters weighing 90±10 g were divided into six diet regimen groups with 10 animals in each group. These regimens were as follows: a control diet consisting of normal food, a HFD, a HFD in combination with one of three PTF-b dosages (50, 100, or 200 mg/kg per day), and a HFD in combination with berberine (200 mg/kg per day). At the end of the 4-week period, after the animals had been fasted overnight, the weight of each animal was measured, and blood samples were collected for the estimation of serum TC, TG, LDL-c and HDL-c (Jian Cheng Biotechnology Company, Nanjing, China). Hamsters were then euthanized, and the weights of the liver and retroperitoneal adipose tissue were determined. The hepatic levels of TC, TG were determined using assay kits (Jian Cheng Biotechnology Company, Nanjing, China) according the manufacturer's instructions. The activity of serum and hepatic LPL was measured by kit (Jian Cheng Biotechnology Company, Nanjing, China) according the manufacturer's instructions.

### Cell culture

HepG2 cells, which were originally from the American Type Culture Collection (ATCC) (Manassas, VA, USA), were obtained from the China Union Medical University. Cells were grown to 70%–80% confluence and then incubated in 0.02% BSA (Sigma-Aldrich)/DMEM (Gibco-BRL, Grand Island, NY, USA) for 12 h. Cells were then washed and incubated with 1 µM of various CQAs or 1 mM of the AMPK activator AICAR (Sigma-Aldrich, China) in 0.02% BSA+100 µM oleic acid/DMEM or in 0.02% BSA+100 µM oleic acid/DMEM alone for 6 h. Subsequently, the cells were subjected to oil-red O staining or TC and TG determination as described previously [30].

### UPLC analysis

The PTF-b (0.01 g) was dissolved in methanol (5 ml×2) under ultrasonic irradiation. The sample was passed through a 0.22 µm filter prior to injection. Known amounts of the reference standards were weighed and dissolved in methanol to prepare solutions with concentrations of approximately 1 mg/ml. The standards were diluted fifty-fold to prepare the working solutions before injection. The samples were separated on an Acquity UPLCTM system (Waters Corp., USA). The chromatographic analysis was conducted on an Agilent phenyl column (100 mm×2.1 mm i.d., 1.7 µm) at 25°C with a flow rate of 0.4 ml/min. The mobile phase consisted of acetonitrile (A) and water with 0.05% formic acid (B), and the eluting gradient was as follows: 0–3 min, 2–11.0%; 3–10 min, 11.0%; 10–16 min, 11.0–20%; 16–18 min, 20–24%; 18–22 min, 24–24%; 22–22.5 min, 24–30%; 22.5–26 min, 30–30%; 26–33 min, 30–80%; 33–35 min, 80–100%.

### Mass spectrometry

Mass spectrometry detection was performed on a Synapt G2 MS system (Waters Corp., USA) equipped with an ESI source. Two data acquisition modes-MS^E^ were selected to investigate precursor ions and product ions. Nitrogen gas was used for nebulization. The detection mode of the flying tube was selected to the “V” pattern. The negative ion spectra of the column eluates were recorded in the range of *m/z* 100–1500. The optimized conditions of the ESI source were as follows: capillary voltage, 2.5 kV; sampling cone voltage, 40 V; extraction cone voltage, 3.0 V; ESI source temperature, 120°C; desolvation temperature, 450°C; cone gas flow, 30 L/h; desolvation gas flow, 800 L/h; Collision gas flow, 0.5 ml/min; collision energy for MS^E^ acquisition mode, 4.0 eV for low energy scan and 15–40 eV for high energy scan; the dynamic adjustment of the fragmentor voltage range was from 25 to 40 V for the MS/MS acquisition mode. The lock mass compound was leucine enkephalin (*m/z* 556.2771), and the interval scan time was 0.02 s. Masslynx 4.1 (Waters Corp.) software was used to control the instrument.

### Oil-Red O staining

Liver tissues of hamsters were removed and each liver lobe was cut into small pieces. To detect fat deposition in the liver, frozen sections were rinsed with distilled water, stained with 0.2% Oil-Red O (Sigma-Aldrich, Shanghai, China) and 60% 2-propanol (Sigma-Aldrich) for 10 min at 37°C, and then rinsed with distilled water. We observed the liver tissues under a microscope (Leica DM4000B, Benshein, Germany) and photographed the samples with a digital camera. The mean gray values of the photos were quantified using the ImageJ 4.1 software.

### NMR-based hepatic metabolomics

The NMR-based hepatic metabolomics including the liver tissue extraction, the pulse-acquire sequence and the metabolite identification, and the data processing was performed according to previous reports with minor modulation [39], [68], [69]. The lipophilic extracts which contained most of the lipid constituents in the liver were used for 1H-NMR spectroscopy. The extracts were mixed with CDCl3 and centrifuged (10,000 rpm, 10 min, 4 °C). The supernatants of each sample were individually transferred into 5-mm o.d. NMR tubes. All 1H-NMR spectra were recorded at 27 °C on a Bruker AVIII 600 spectrometer (Bruker Biospin, Germany) equipped with an inverse 5-mm Bruker probe operating at a 600.13 MHz 1H frequency. A simple 90° pulse-acquire sequence was used, and total 128 transients were collected into 32768 data points, with a relaxation delay of 2 s and an acquisition time of 2.65 s.

The acquired NMR spectra were referenced to the chemical shift of CDCl_3_. Following phase and baseline correction, the ^1^H-NMR spectra were automatically reduced to ASCII files using the AMIX software (Analysis of MIXtures software v. 3.0, Bruker Biospin). Each spectral region was normalized to the total of all the resonance integral regions and reduced to ‘buckets’ of equal width (0.01 ppm) over the range of 0.5–6.0 ppm. The generated ASCII files were imported into SIMCAP 12.0 (Umetrics, Umeå, Sweden) for the PR analysis. Prior to the analysis, the values of all variables were mean-centered. The metabolites in the liver extracts were identified with reference to the published literature data [39] and using the Chenomx NMR Suite (Chenomx, Calgary, Canada).

### Real-time quantitative PCR

The mRNA levels of twenty-one lipid metabolism-related genes were determined by real-time quantitative PCR. Total RNA extraction, cDNA synthesis and quantitative PCR assays were performed as described previously [70], [71]. The normalized expression levels of the target genes were estimated as described previously [72]. At least three independent biological replicates were performed to check the reproducibility of the data. The gene-specific primers used for quantitative PCR are listed in Table S2.

### Western blotting

Livers and retroperitoneal adipose tissue were lysed in lysis buffer containing 10% glycerol, 1% Triton X-100, 135 mM NaCl, 20 mM Tris (pH 8.0), 2.7 mM KCl, 1 mM MgCl2, and protease and phosphatase inhibitors (0.5 mM PMSF, 2 µM pepstatin, and 2 µM okadaic acid). The protein concentrations were assessed using the Bradford method, and aliquots of samples containing 40 µg of protein were subjected to SDS-PAGE (12% and 7.5% for P-AMPK and P-ACC, respectively) and then transferred to PVDF membranes (Amersham Pharmacia, Uppsala, Sweden). Immunoblotting was performed using phospho-AMPKα (Thr-172, 1∶3000) and phospho-ACC (1∶2000) antibodies (Cell Signaling Technology, Beverly, MA, USA), as well as anti-beta-actin (included as a loading control; Abcam, Inc., Cambridge, MA, USA). Following incubation with horseradish peroxidase-conjugated secondary antibodies (Sigma-Aldrich, China), the proteins were detected with an ECL plus kit (Amersham, Piscataway, NJ, USA). Immunoreactive bands were quantified using NIH image analysis software.

### Statistical analyses

Data are presented as the means ± S.E.M. One-way ANOVA was used to determine significant differences among groups, after which the modified Student's t-test with the Bonferroni correction was used for comparison between individual groups. P<0.05 was considered statistically significant.

## Supporting Information

Figure S1 **The CQA-rich PTF-b reversed some hyperlipdemia-related metabolites.** (**A**) ^1^H-NMR peak attribution of the main metabolites in the liver chloroform extract from the hyperlipidemic hamster. Visual comparison of the ^1^H-NMR spectra between the HFD group and normal (**B**), HFD+berberine (**C**) or HFD+PTF-b (**D**) groups displayed apparent changes in 8 signals. No visual differences were detected between the HFD+PTF-b and the HFD+berberine group (**E**). Keys: (**1**) Cholesterol (Me-18), (**2**) methyl groups of fatty acid and cholesterol, (**3**) Cholesterol (Me-19), (**4**) fatty acid residue (CO-CH_2_-CH_2_), (**5**) fatty acid residue (-CH_2_-CH = ), (**6**) fatty acid residue (-CO-CH_2_), (**7**) N^+^(CH_3_)_3_ (PC and SM), (**8**) fatty acid residue (-CH = CH-).
(TIF)

Figure S2 **Statistical analysis of the apparently changed lipid signals by one-way ANOVA showed significant difference among the normal, HFD, HFD+berberine and HFD+PTF-b groups.** **p<0.01, ***p<0.001 HFD group vs. normal group. ^†^ *p*<0.05, ^‡^ *p*<0.01 test group vs. HFD group.
(TIF)

Table S1 **List of the caffeoylquinic acid derivatives identified in the** ***n*** **-butanol fraction of** ***P. tectorius*** **(PTF-b) by their retention times, UV and mass spectra and by comparison with published data or commercial standards.**
(DOC)

Table S2 **Oligonucleotide primers used in this work.**
(DOC)
