# Supplementary figures and images for: Anti-Hyperlipidemic Effects and Potential Mechanisms of Action of the Caffeoylquinic Acid-Rich Pandanus tectorius Fruit Extract in Hamsters Fed a High Fat-Diet
Source: PLoS One. 2013 Apr 16;8(4):e61922. doi: 10.1371/journal.pone.0061922 (PMC3628350; doi:10.1371/journal.pone.0061922)

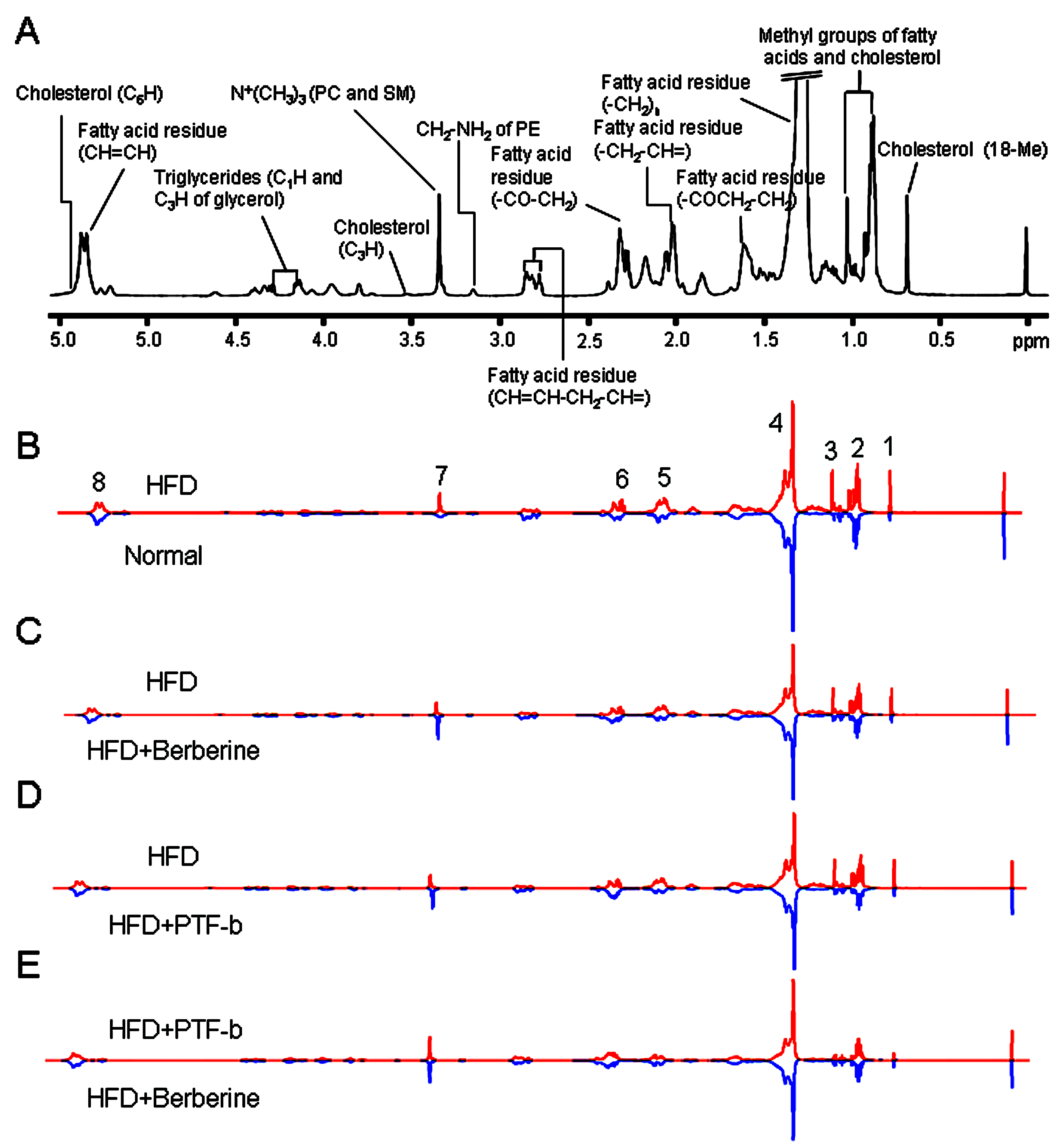

Supplement: Figure S1 — The CQA-rich PTF-b reversed some hyperlipdemia-related metabolites. (A) 1H-NMR peak attribution of the main metabolites in the liver chloroform extract from the hyperlipidemic hamster. Visual comparison of the 1H-NMR spectra between the HFD group and normal (B), HFD+berberine (C) or HFD+PTF-b (D) groups displayed apparent changes in 8 signals. No visual differences were detected between the HFD+PTF-b and the HFD+berberine group (E). Keys: (1) Cholesterol (Me-18), (2) methyl groups of fatty acid and cholesterol, (3) Cholesterol (Me-19), (4) fatty acid residue (CO-CH2-CH2), (5) fatty acid residue (-CH2-CH = ), (6) fatty acid residue (-CO-CH2), (7) N+(CH3)3 (PC and SM), (8) fatty acid residue (-CH = CH-). (TIF) [file pone.0061922.s001.tif]

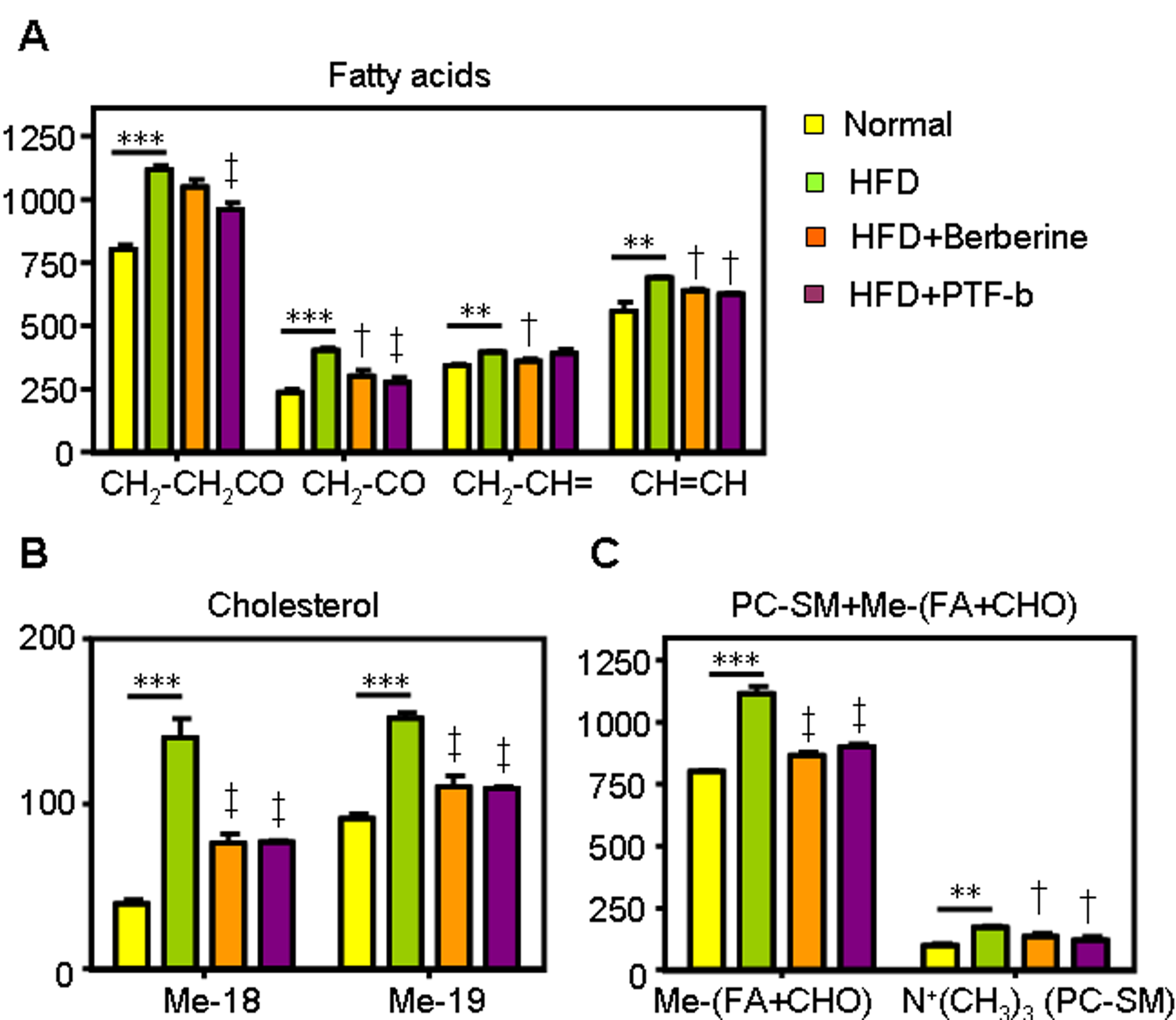

Supplement: Figure S2 — Statistical analysis of the apparently changed lipid signals by one-way ANOVA showed significant difference among the normal, HFD, HFD+berberine and HFD+PTF-b groups. **p<0.01, ***p<0.001 HFD group vs. normal group. † p<0.05, ‡ p<0.01 test group vs. HFD group. (TIF) [file pone.0061922.s002.tif]
