# Supplementary material for: Anti-Hyperlipidemic Effects and Potential Mechanisms of Action of the Caffeoylquinic Acid-Rich Pandanus tectorius Fruit Extract in Hamsters Fed a High Fat-Diet
Source: PLoS One. 2013 Apr 16;8(4):e61922. doi: 10.1371/journal.pone.0061922 (PMC3628350; doi:10.1371/journal.pone.0061922)
Supplement: Table S1 — List of the caffeoylquinic acid derivatives identified in the n -butanol fraction of P. tectorius (PTF-b) by their retention times, UV and mass spectra and by comparison with published data or commercial standards. (DOC) [file pone.0061922.s003.doc]

Table S1. List of the caffeoylquinic acid derivatives identified in the *n*-butanol fraction of *P. tectorius* (PTF-b) by their retention times, UV and mass spectra and by comparison with published data or commercial standards.

| #Peak | Chemical name | R_1_ | R_3_ | R_4_ | R_5_ | R_6_ | Absorbance maxima (nm) | [M-H]^-^ | Fragments and their intensity (%) |
| --- | --- | --- | --- | --- | --- | --- | --- | --- | --- |
| 1 | 1-O-caffeoylquinic acid | C | H | H | H | H | 327 | 353.0872 | 191.0557 (100), 179.0356 (29.1), 135.0444 (21.3) |
| 2 | 3-O-caffeoylquinic acid | H | C | H | H | H | 326 | 353.0880 | 191.0557 (88.7),179.0356 (84.3), 173.0448 (100), 135.0446 (63.2) |
| 3 | 4-O-caffeoylquinic acid | H | H | C | H | H | 325 | 353.0872 | 191.0558 (100) |
| 4 | 5-O-caffeoylquinic acid | H | H | H | C | H | 326 | 353.0888 | 191.0557 (100), 161.0242 (15.0) |
| 5 | 1, 3-di-O-caffeoyl-*epi*-quinic acid | *EQ* | *EQ* | H | H | H | 326 | 515.1172 | 353.0890 (97.6), 191.0560 (68.7) 179.0354 (100), 173.0453 (77.3) |
| 6 | 3, 4-di-O-caffeoyl quinic acid | H | C | C | H | H | 327 | 515.1189 | 353.0881 (88.1), 191.0555 (47.2), 179.0338 (87.8), 173.0445 (100) |
| 7 | 3, 5-di-O-caffeoyl-*epi*-quinic acid | H | *EQ* | H | *EQ* | H | 325 | 515.1196 | 353.0881 (100), 191.0555 (52.5), 179.0338 (73.8), 173.0445 (86.3) |
| 8 | 1, 3-di-O-caffeoylquinic acid | C | C | H | H | H | 327 | 515.1196 | 353.0881 (84.2), 191.0555 (100), 179.0338 (64.1), 173.0445 (24.8) |
| 9 | 3, 5-di-O-caffeoylquinic acid | H | C | H | C | H | 326 | 515.1205 | 353.0881 (94.5), 191.0555 (100), 179.0338 (51.8), 173.0445 (11.9) |
| 10 | 1, 5-di-O-caffeoylquinic acid | C | H | H | C | H | 327 | 515.1179 | 353.0881 (100), 191.0555 (39.3), 179.0338 (67.4), 173.0445 (71.7) |
| 11 | 4, 5-di-O-caffeoylquinic acid | H | H | C | C | H | 326 | 515.1198 | 353.0881 (100), 191.0555 (32.8), 179.0338 (63.0), 173.0445 (81.8) |
| 12 | Methyl 1, 3-di-O-caffeoyl quinate | H | C | C | H | Me | 327 | 529.1350 | 515.1189 (11.6), 367.1029 (100), 179.0346 (22.3), 161.0242 (99.0) |
| 13 | 1, 4-di-O-caffeoylquinic acid | C | H | C | H | H | 326 | 515.1191 | 353.0881 (100), 191.0555 (48.2), 179.0338 (68.2), 173.0445 (83.3) |
| 14 | 3, 4, 5-tri-O-caffeoyl quinic acid | H | C | C | C | H | 325 | 677.4943 | 529.1385 (100), 367.1029 (66.8), 353.0868 (22.7), 174.9570 (87.8) |
| 15 | Methyl 3, 4-di-O-caffeoyl quinate | H | H | C | C | Me | 327 | 529.1354 | 367.1039 (78.1), 179.0348 (100), 161.0242 (46.2), 135.0457 (36.7) |
