# Supplementary material for: Anti-Hyperlipidemic Effects and Potential Mechanisms of Action of the Caffeoylquinic Acid-Rich Pandanus tectorius Fruit Extract in Hamsters Fed a High Fat-Diet
Source: PLoS One. 2013 Apr 16;8(4):e61922. doi: 10.1371/journal.pone.0061922 (PMC3628350; doi:10.1371/journal.pone.0061922)
Supplement: Table S2 — Oligonucleotide primers used in this work. (DOC) [file pone.0061922.s004.doc]

Table S2. Oligonucleotide primers used in this work.

| Primers | Sequence (5’-3’) |
| --- | --- |
| ACC-F | ACACTGGCTGGCTGGACAG |
| ACC-R | CACACAACTCCCAACATGGTG |
| SCD-F | ccgagagacttcagggaacttg |
| SCD-R | ggtaggcaggtatgctccga |
| PPARα-F | GAGAAAGCAAAACTGAAAGCAGAGA |
| PPARα-R | GAAGGGCGGGTTATTGCTG |
| PPARγ-F | CACTCCCATTCCTTTGACATCA |
| PPARγ-R | AGCTGGGCCTTTTCAGAATAATAA |
| apoB-F | TGCTCCCATTTTAACAATGAAC |
| apoB-R | TAAACTTGTGGTCAACACCTCC |
| SREBP-1a-F | GGCTGTGGAACAGGCACTG |
| SREBP-1a-R | AGCTGGAGCATGTCTTCGATG |
| SREBP-1c-F | GCGGACGCAGTCTGGG |
| SREBP-1c-R | ATGAGCTGGAGCATGTCTTCAAA |
| SREBP-2-F | cgttctgaggaaggccattg |
| SREBP-2-R | agcaccatgttctcctgacgta |
| ABCA1-F | gggacacacaggcagcatc |
| ABCA1-R | aatgaagaacccctcgccc |
| MTP-F | gggtcctcttccgcctatactg |
| MTP-R | aataaagcatgtcgaggctgtatg |
| FAS-F | AGCCCCTCAAGTGCACAGTG |
| FAS-R | TGCCAATGTGTTTTCCCTGA |
| LDLR-F | GATCCCCAACCTGAAGAACG |
| LDLR-R | AGTCTACGGCCAGCCCATCT |
| LPL-F | CAGCTGGGCCTAACTTTGAG |
| LPL-R | CCTCTCTGCAATCACACGAA |
| LXR-α-F | GCAACTCAATGATGCCGAGTT |
| LXR-α-R | CGTGGGAACATCAGTCGGTC |
| HMGR-F | GCCGGCAGCTCCTCTCCAC |
| HMGR-R | AGCCTTCCGTTGTTGCCATTG |
| ACO-F | CCTTCCTGCTCACACACTTCTG |
| ACO-R | TGGTATAACTGCTGCCATCATAGG |
| HSL-F | GGTGACACTCGCAGAAGACAATA |
| HSL-R | GCCGCCGTGCTGTCTCT |
| CPT1-F | CTCAGTGGGAGCGACTCTTCA |
| CPT1-R | GGCCTCTGTGGTACACGACAA |
| Insig2-F | CCAAGGATCCATGCCCAGGCTGCACGACCAC |
| Insig2-R | CCAAGCGGCCGCTCAGTCACTGTGAGGCTTTTCCGG |
| SCAP-F | GATGTCCATTGTCTTTGGTATCC |
| SCAP-R | AGACCGTCCTCTTCCTTGTG |
| CYP7A1-F | GGTAGTGTGCTGTTGTATATGGGTTA |
| CYP7A1-R | ACAGCCCAGGTATGGAATCAAC |
| β-Actin-F | TCTACAATGAGCTGCGTGTG |
| β-Actin-R | GGTCAGGATCTTCATGAGGT |
